# Vaginal Examinations and Vaginal Douches in Normal Labor

**Published:** 1898-12

**Authors:** 


### VAGINAL EXAMINATIONS AND VAGINAL DOUCHES IN NORMAL LABOR.

*Medical Record*, September 17, 1898: From a paper by George P. Shears, M. D., we extract as follows: A brief survey of recent progress in the bacteriology of obstetrics will suffice to show what might have been expected at the outset, namely, that in normal cases we can not improve upon the methods of nature. It has been clearly shown that the vaginal secretions have bactericidal properties, and that in normal cases antiseptic douches can only aid in causing infection. Since, however, the non-pathogenic character of the organisms normally found in the vagina has been demonstrated such measures as preliminary douching vaginal, scrubbing in normal cases, and the routine use of daily post-partum douches have been very generally abandoned, and in normal cases we have as obstacles to perfect asepsis only frequent vaginal examinations still often practiced, and the post-partum douches still used by many practitioners.

To what extent can we dispense with these? First, we note that in normal labor no douches of any kind are necessary. But what is normal labor? Labor complicated by vaginal examinations and douches is not normal. How beautifully has nature provided against infection! Not only are the vaginal secretions antagonistic to the germs of infection, but after rupture of the membranes the cervico-vaginal canal is flooded with liquor amnii, which some one has not inaptly called a normal salt solution, and the fœtus during its emergence is at all times closely embraced by the retracting ostium vaginæ, so that neither air nor foreign body can enter. Here there is clearly no indication for the post-partum douche, unless, indeed, some enterprising person has given an ante-partum douche. Since sepsis comes from without, and chiefly through vaginal examinations, the question naturally arises, why take the chances of introducing sepsis at all? It would, of course, be a dangerous absurdity to endeavor to prohibit vaginal examination in labor, but that it is vastly overdone there can be no possible doubt. It is difficult to understand why more stress is not laid upon the admitted importance of external palpation. The fact that surgeons of experience disagree as to the best method of hand disinfection confirms the conclusion of modern bacteriologists that no methods are infallible.

When a delayed first stage threatens the life of mother or child, it is necessary to introduce the hand into the uterus for the purpose of making an exact diagnosis, if the diagnosis can be made in no other way. But who would advise this as a routine practice? The day is not distant when it will be generally acknowledged that it is no less an absurdity to make frequent vaginal examinations in normal cases, especially when the finger is passed through and around the partially dilated cervix, in order to determine the progress of labor. The danger of sepsis is distinctly increased when the finger is carried within the cavity of the cervix, or into the lower urine segment, where the acid vaginal mucus no longer aids in inhibiting bacterial development. The term meddlesome midwifery should not be applied to obstetric operations, which, when indicated, are really conservative measures, but rather to unwarranted interference with normal labor, and especially to examinations and douches that are not necessary. It is as futile to interfere with nature's process in normal labor as to rely upon her unaided efforts in abnormal labor.

While unnecessary examinations are objectionable before delivery, they are doubly so afterward. To distend the cervico-vaginal canal in search of small tears, whose edges are in apposition, and have no tendency to separate, would be ludicrous if it were not so dangerous. The practice of introducing the hand into the uterus to remove fragments of membrane which have been torn off during the delivery of the placenta, should be unhesitatingly condemned as far more dangerous than allowing them to remain.

To sum up, the common custom of making frequent vaginal examinations during the whole course of labor is unscientific and unsafe, and the obstetrician should so familiarize himself with all extra-vaginal methods for diagnosis, as to reduce the necessity for vaginal examinations to a minimum. All intra-vaginal manipulations are especially objectionable after delivery.

When the vaginal exeretions are normal, ante-partum douches are unnecessary and harmful. In normal cases the single post-partum douche is unnecessary, and, therefore, objectionable, but in many cases circumstances render it advisable as a precautionary measure. The routine use of douches during the normal puerperium is contraindicated.

Since the memorable reform of Garrigues at the New York Maternity, deaths from puerperal sepsis have been comparatively rare among us, but they are by no means unknown, while the minor degrees of sepsis occur all too often, and furnish our gynecologists with much employment. Nor will this condition of affairs be remedied until it is generally recognized that in normal labor the vagina is no more a legitimate field for experiment than the uterus.—*Medical Fortnightly.*

